# A Robot-Operation-System-Based Smart Machine Box and Its Application on Predictive Maintenance

**DOI:** 10.3390/s23208480

**Published:** 2023-10-15

**Authors:** Yeong-Hwa Chang, Yu-Hsiang Chai, Bo-Lin Li, Hung-Wei Lin

**Affiliations:** 1 Department of Electrical Engineering, Chang Gung University, Taoyuan City 333, Taiwan; 2 Department of Electrical Engineering, Ming Chi University of Technology, New Taipei City 243, Taiwan

**Keywords:** robot operating system, machine box, predictive maintenance, machine learning

## Abstract

Predictive maintenance is a proactive approach to maintenance in which equipment and machinery are monitored and analyzed to predict when maintenance is needed. Instead of relying on fixed schedules or reacting to breakdowns, predictive maintenance uses data and analytics to determine the appropriate time to perform maintenance activities. In industrial applications, machine boxes can be used to collect and transmit the feature information of manufacturing machines. The collected data are essential to identify the status of working machines. This paper investigates the design and implementation of a machine box based on the ROS framework. Several types of communication interfaces are included that can be adopted to different sensor modules for data sensing. The collected data are used for the application on predictive maintenance. The key concepts of predictive maintenance include data collection, a feature analysis, and predictive models. A correlation analysis is crucial in a feature analysis, where the dominant features can be determined. In this work, linear regression, a neural network, and a decision tree are adopted for model learning. Experimental results illustrate the feasibility of the proposed smart machine box. Also, the remaining useful life can be effectively predicted according to the trained models.

## 1. Introduction

In recent years, with the vigorous development of Internet of Things (IoT) technologies, everything connected everywhere has become a vision just around the corner. Fueled by this wave, the industry demands for smart factory automation are also increasing [1,2,3,4]. Smart manufacturing integrates manufacturing resources with sensors, computing platforms, and communication technology. Recently, data-driven decision making has attracted much attention [5,6,7]. In general, the growth of production efficiency and the improvement in product quality rely closely on the automation equipment. To expand production capacity, enterprises generally account for a considerable proportion of investment in purchasing new equipment. The equipment reliability becomes more crucial to ensure the normal production process. Most of the maintenance mechanisms stay in the so-called prevention maintenance realm. Practically, the overall equipment effectiveness is related to the length of use, operation mode, and equipment quality, as well as the environmental factor and other uncontrollable factors. In many cases, there is no unique maintenance rule, even if the equipment comes from the same batch number. Under the fixed-schedule maintenance mode, if the equipment suddenly fails, it often causes a great influence such as the wasting of manpower and the increase in costs. In another way, predictive maintenance (PdM) extracts the crucial features from the physical parameters to produce a health indicator through a modelling and validation process [8,9,10,11]. In general, the typical parameters of manufacturing automation equipment include the vibration, sound noise, temperature, voltage and current, etc. Following the process of predictive maintenance, the remaining useful life of equipment can be predicted such that the necessary treatment can be performed in advance. To improve the operation effectiveness of factory automation, different PdM solutions have been proposed. For example, Microsoft developed a predictive maintenance solution based on the Azure AI platform to solve some business problems. German software company SAP launched a PdM solution for an equipment pre-diagnosis to improve the utilization rate. A similar experience involves a Japanese automobile company—50% of failures can be predicted 2 h in advance, which saves 1.5 h per failure with the use of advanced prediction. 

A smart machine box (SMB) is a data capture device used to interface with automation equipment. The collected data can be used for a subsequent data analysis such as feature extraction, modeling, and verification, and then provides decisions that can help to optimize production efficiency. In response to the global development trends, Taiwan has also put forward many policies that are helpful for industrial upgrading. For example, the IoT information security industry standards were promoted in 2017, and they became national standards in 2019. In addition, a development plan, from 2017 to 2025, of digital country and economy innovation has been stimulated by the government. Also, a subsidy training course for the SMB technology and application was provided by the Industrial Bureau of the Ministry of Economic Affairs. These policies are mainly aimed at assisting the domestic manufacturing industry to bring in networked equipment, implement production management visualization and intelligence, and enhance their international competitiveness. In summary, the efficiency improvement in automation industries has attracted much attention globally. The sensor installation and operation, data capturing and analysis, and the model learning with algorithms are helpful to know the ongoing status of equipment better. 

In industrial applications, there are some successful studies of using sensing technology, data fusion, a correlation analysis, health indicators, and other concepts. Versatile applications can be found in the literature [12,13,14,15,16,17,18,19,20,21,22,23]. For examples, SVM was used to estimate the remaining life of stepper motors [12]. A machine learning approach was applied to predict the maintenance status of wafer stick machines [13]. LSTM and SVR were used to estimate the remaining life of an engine [14]. In [15], a decision fusion method was proposed that is superior to the methods with an individual sensor in terms of prediction accuracy and stability. In [16], a fuzzy inference network model was proposed for bearings and gears. Furthermore, some degradation features taken from bearing vibration signals were performed with PCA dimensionality reduction, where the remaining life was analyzed [17]. In [18], a gray correlation analysis was used to estimate the remaining useful life (RUL) of battery capacity. The remaining life of milled tools was predicted with the method of the autoregressive comprehensive moving average [19]. In the work of [20], an adversarial network was proposed to predict remaining useful life, where two open data sets, CMAPSS and FEMTO, were used. Moreover, a framework for predictive maintenance in an injection molding process was presented [21]. In the application of batteries, a predictive model was proposed, where the remaining useful life of lithium-ion batteries can be predicted [22]. Some vibration sensors were applied to monitor the running status of centrifugal pumps, such that the mechanical and fluid faults can be diagnosed [23]. 

The priority to smart manufacturing is to gather machine operating states. A smart machine box is a device mainly consisting of a computation core, connecting interfaces, and a possible wireless communication module [24,25,26]. In [25], Raspberry Pi is chosen as the core platform to build the smart machine box. This study aims to propose a predictive maintenance integration scheme. The development of a smart machine box is helpful for the allocation and construction of a sensing environment while also establishing autonomous ability for data capture and transmission. The subsequent data analysis process such as data preprocessing, a feature analysis, modeling, and prediction can clearly present the health conditions of automation equipment. In this work, the key characteristics of the created smart machine box will be described. Also, an integrated experiment is conducted, where an air filtering system is considered for the data sensing and the verification of health status prediction. The main contributions of this paper are listed as follows:

1. A smart machine box is designed and implemented with versatile communication interfaces that can be adopted to different sensor modules.
2. Based on the ROS platform, the data collection, transmission, and processing can be performed individually in a multiplexed manner, and the function of hot swapping will be more efficient.
3. Integrated with the proposed smart machine box, experimental tests are performed to validate the feasibility on the application of predictive maintenance.
4. The key concepts of data preprocessing, a feature analysis, model training, and validation are discussed in detail, and the remaining useful life of the experimental system can be effectively obtained.

## 2. Materials and Methods

In general, the voltage, current, position, speed, and other working states of physical systems can reflect whether the system is properly operated. Through the analysis of captured physical phenomena, the operational efficiency of the system can be effectively identified. In the predictive maintenance of networked devices, the perception of the physical parameters depends on the installation of appropriate sensors combined with an embedded computing core. Also, versatile communication interfaces could be necessary to meet the multi-purpose applications of interest. Finally, the collected data will be used for prediction of remaining useful life by using some machine learning methods. 

### 2.1. Embedded Computing Core

Considering the factors of a small size, a low price, and necessary functions, a cost–effect embedded computing core includes microcontrollers (MCUs) and a single-board computer. Typically, the MCU has strong peripheral capabilities that are suitable to communicate with external sensor modules. The single-board computer can install a compatible operating system, and the capability could be affordable for reasonable computation load [27,28,29]. In this work, the computing core integrated with MCUs and a single-board computer is presented for the design of smart machine boxes. Common single-board computers include Raspberry Pi, Banana Pi, and Orange Pi, etc., in which Raspberry Pi has more complete supports. According to the public discussion, the Raspberry Pi is relatively stable with more newly released technologies. Raspberry Pi was conceived to be a complete working computer. Also, the other all-in-one devices are more expensive than Raspberry Pi. 

### 2.2. Data Transmission

There are a variety of sensing modules, in which an inter-integrated circuit (I2C), serial peripheral interface (SPI), analog-to-digital converted (ADC), and universal asynchronous receiver/transmitter (UART) are typical communication interfaces. UART is an asynchronous serial communication interface with the communication distance up to 15 m. Considering a longer communication distance, a higher transmission rate, or more networked devices, RS-232, RS-422, and RS-485 are compatible protocols to UART with some converting chips.

RS-232 is designed for a point-to-point communication, the communication distance between devices is not more than 15 m, and the transmission rate is up to 20 kB/s. Therefore, RS-232 is suitable for communication between local devices. In RS-485 communication, the data signal adopts the differential transmission mode, and the RS-485 bus standard is widely used when the communication distance is required to be tens of meters to thousands of meters. RS-485 rejects common-mode interference and is highly sensitive to detect voltages as low as 200 mV. RS-485 operates as a half-duplex, which is convenient for multipoint interconnects and eliminates many signal lines. The application of RS-485 can be networked to form a distributed system, which allows up to 32 drivers and 32 receivers to be connected in parallel. Differential data transmission is the fundamental reason for the long transmission distance under the same rate conditions, which is the fundamental difference between RS-422, RS-485, and RS-232. Basically, RS-232 transmits messages in a half-duplex manner, RS-422 can be sent and received in a full-duplex manner through two pairs of twisted pairs, and RS-485, although only a half-duplex, only requires a pair of twisted pairs. RS-422 and RS-485 can transmit 1200 m at 19 Kb/s.

### 2.3. Data Processing and Analysis

In practical applications, the raw data captured with the sensor need to go through the preprocessing process such as missing-data imputation, normalization, and standardization. There are many reasons that cause missing data. For examples, data could be missing during transmission, or sensor noise could exist. Commonly used solutions include relacing the missing data with averaged values or linear interpolated values. Data normalization involves transforming the data into a standard format or scale to make them more suitable for an analysis or modeling. Normalizing data ensures that variables with different units and scales can be compared directly. The normalization process can avoid the computation bias between data fields. A data analysis mainly includes the correlation analysis and feature analysis. In the process of correlation, it is desired to fine the correlation between features, where Pearson, Spearman, and Kendall correlations are commonly considered. In general, features can be represented as time- and frequency-domain features. Typical features of data include the mean, standard deviation, skewness, kurtosis, etc. Through the correlation analysis, the dominant features can be extracted such that the computation load for the following modeling and verification can be reduced. The model of the system can equivalently represent the dynamic relationship of the system, which can effectively predict the change trend of the system. Basically, the system model can be derived through an input and output data analysis. In general, machine learning methods are applied to build the system model. Typical machine learning algorithms include a neural network, linear regression, a decision tree, etc. 

## 3. Results

### 3.1. ROS Framework

**Figure 1:**
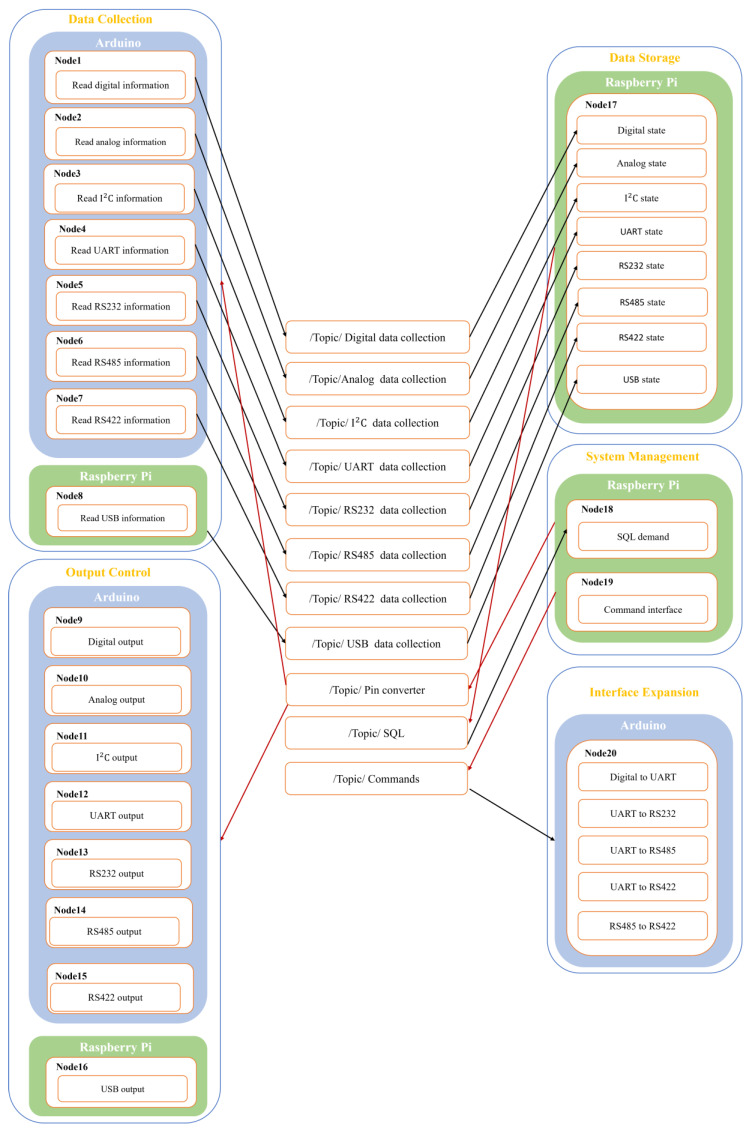
ROS framework of smart machine box.

In the Robot Operation System (ROS) framework, the so-called message, first delivered to the topic, is transferred from one node to another node. The topic is similar to a bulletin board where nodes post their messages and each node has free access. The node that sends a message is called a publisher, and the node that receives a message is called a subscriber [30,31,32,33]. The ROS-based framework is really flexible and adaptable to the needs of the user. In this study, the system integration, including the data sensing and interface management, is based on a ROS framework as shown in Figure 1. In this ROS framework, the whole system is divided into five packages, namely data collection, output control, data storage, system management, and interface expansion. In the ROS framework, each node transmits or receives data to/from other nodes through topics by acting as a publisher or subscriber. For example, the sensing data of Nodes 1~8 were published, and the information can be received by Node 17 by subscribing to topics. Then, the data in the data storage package publish messages to an SQL topic. Then, Node 18 can receive the message by subscribing. This is the whole process from sensing to remote storage. Under the ROS framework, each node can perform one-to-one, one-to-many, many-to-one, and many-to-many data sharing regardless of a publisher or a subscriber. The advantage of writing a program under the ROS framework is that the program execution of all nodes can be performed separately in a multiplexed manner.

### 3.2. Smart Machine Box

**Figure 2:**
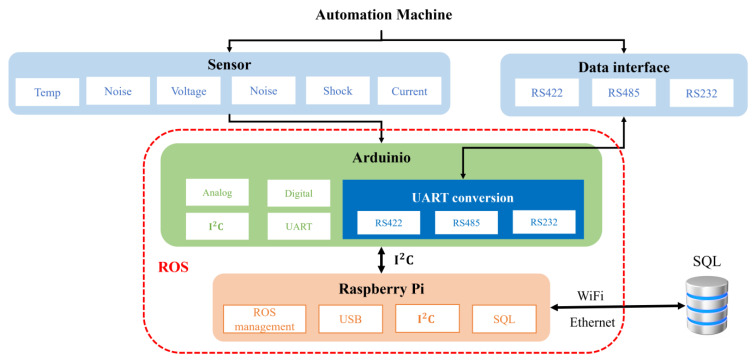
Scheme diagram of the smart machine box.

The smart machine box, having a variety of communication interfaces, is responsible for the front-end data collection. The proposed SMB supports the hot-swap function such that sensor modules can be installed/uninstalled online. With the multi-function computing core, wireless communication can be achieved with high efficiency and low power consumption. Also, the flexible and scalable communication configuration can afford interface adjustment according to task demands of concern. The scheme diagram of the proposed smart machine box is shown in Figure 2. The physical information from sensors can be extracted with Arduino. Also, the states of an automation machine can be received through the communication interfaces, RS232, RS422, and RS485. The communication interface between Arduino and Raspberry Pi is $I^{2}C$. The management of the ROS framework is built in Raspberry Pi that is also responsible for the remote data storage.

In addition to general-purpose input/output (GPIO), the on-board communication interfaces include UART, USB, and HDMI. HDMI can be used to connect a high-resolution monitor. Either UART or USB can be converted into RS232, RS422, or RS485 with some converter modules. In the short-distance applications, GPIOs, RS232, and USB are appropriate to use, for example, when the distance is less than 2 m. As for the applications in a large-scale factory, the distance between interface ports is very likely tens of meters or more. In these cases, RS422 or RS485 is a good choice to avoid signal attenuation. Furthermore, the concept of hot swapping is considered to improve the installation convenience of sensor modules. With the hot swapping, there is no need to shut down/restart the SMB or do any pin setting. If there are some interfaces available, one can easily link the sensor module to the available communication port if the physical characteristics match. 

**Figure 3:**
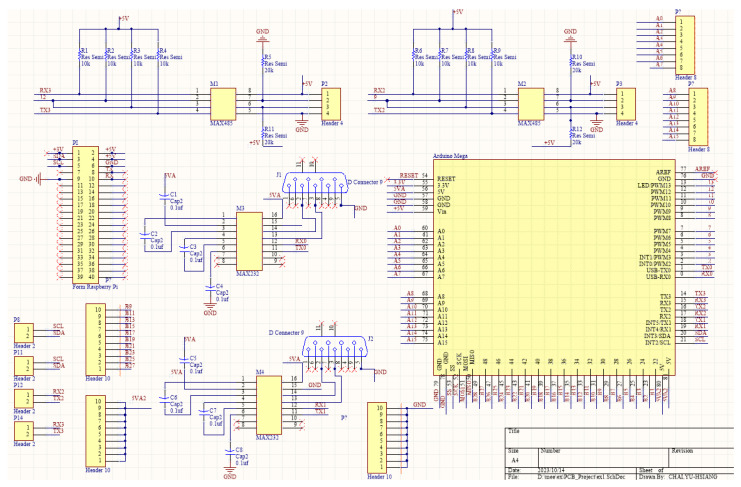
Circuit design layouts of the SMB.

**Figure 4:**
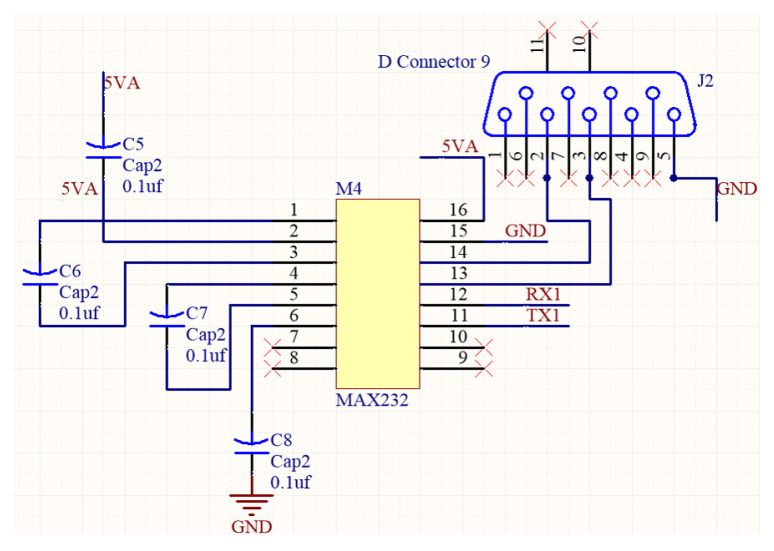
Circuit for RS232 to UART.

**Figure 5:**
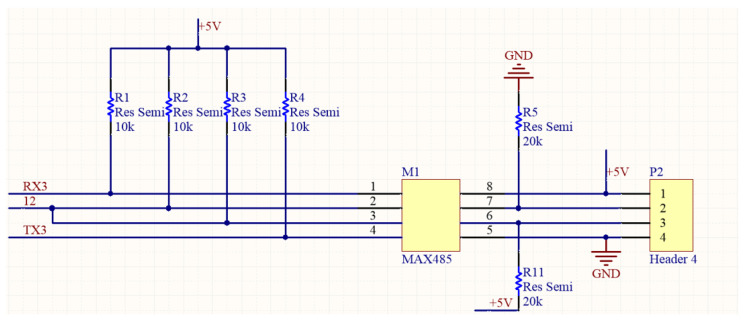
Circuit for RS485 to UART.

The computing core is mainly composed of Arduino and Raspberry Pi boards. Arduino Mega 2560 is used here as a microcontroller module that is responsible for the data extraction from sensors. On the other hand, Raspberry Pi 3B is mainly used for process management and sending the processed data to the database. The AutoCAD circuit diagram of the proposed machine box is shown in Figure 3. The major sub-circuits in Figure 3 include the pin assignment of Arduino Mega 2560, the connection interface of Raspberry Pi, and the signal conversion circuits between UART and RS232/RS485. It can be seen that there are two sets of RS232 to UART conversion interfaces. Also, there are two sets of RS485 to UART. The UART is a popularly serial interface that consists of two pins, TX and RX. The RX pin is used to receive data, and the TX pin is used to transmit data. When two devices are connected using a UART, the RX pin of one device is connected to the TX pin of the second device. In Figure 4, the RS232 D-sub connector is connected to a MAX232 driver/receiver device, and then the TX/RX can be obtained as the pins TX1 and RX1. In Figure 5, the signal data and clock of an RS485 connector are sent to a MAX485, and then the TX/RX can be obtained as the pins TX3 and RX3.

**Figure 6:**
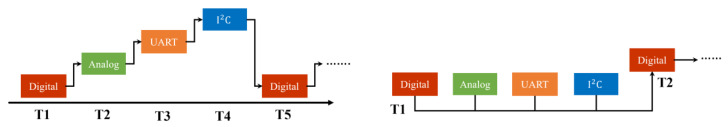
Concept representation of ROS-based hot swapping (regular case).

**Figure 7:**
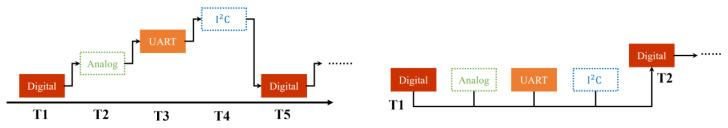
Concept representation of ROS-based hot swapping (unplugging some sensors).

For a computer system, hot swapping or hot plugging is the replacement or addition of components without stopping, shutting down, or rebooting the system. In the developed smart machine box, hot swapping means connecting or disconnecting a sensor module without stopping or shutting down the machine box. Based on the ROS platform, the hot-swapping scenarios will be more efficient. In Figure 6, device pooling is sequential without ROS. Thus, the system without ROS needs four time steps to complete the pooling per cycle. Alternatively, only one time step is required to complete all sensor module pooling per cycle. In another case, if the analog and $I^{2}C$ sensor modules are removed from the communication interface, the mechanism without ROS needs four time steps to recognize this situation, shown as Figure 7. However, the ROS-based device needs one time step to identify the real-time situation.

### 3.3. Predictive Maintenance

**Figure 8:**
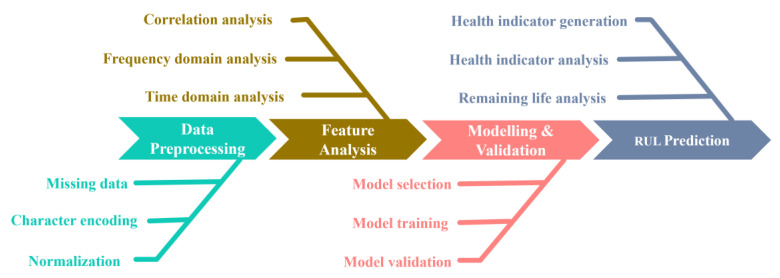
Analysis procedures of predictive maintenance.

This work uses MATLAB to build a predictive maintenance system. Through the data analysis process, the remaining life of the equipment can be predicted, so that the waste of manpower and cost can be reduced. Predictive maintenance is an approach of investigating potential equipment malfunctions to prevent failures in real time. This enables an automated factory to optimize maintenance scheduling and improve reliability. The content of predictive maintenance includes data preprocessing, a feature analysis, modelling, and verification. Finally, the remaining useful life can be predicted through the health indicator and predicted outcomes, shown as Figure 8. 

In general, the execution of missing data and normalization is conducted in the data preprocessing stage. Linear interpolation is one common way to fix the missing data. On the other hand, normalization is the data scaling from the source data into the interval [0, 1] without changing its original distribution. In the feature analysis, a correlation analysis is mainly considered. A correlation analysis provides some quantitative ways to give us a clearer picture of the data itself. For example, a Pearson correlation coefficient can measure the degree of linear dependence between two sets of data. In this process, the features with high correlation coefficients will be chosen as the dominant features for a consequent modeling procedure. The imported data for modelling and validation will be divided into a training set and validation set. In this work, linear regression, a neural network, and a decision tree are adopted for model training. In the model validation stage, the validation data will be sent into the trained model, where the error analysis will be performed to identify which learning model is better. Finally, the best model selected from the modelling and validation process will be applied to predict the remaining useful life. In general, there is a confidence interval to indicate the distribution of the estimated outcomes. 

### 3.4. Integration Test

**Figure 9:**
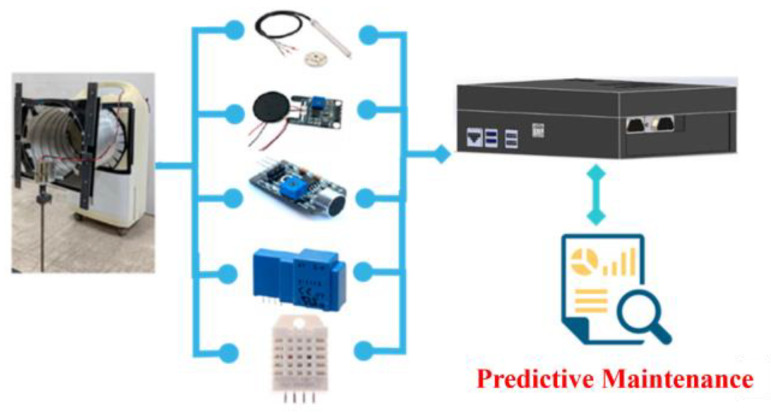
Experimental scheme for integration test.

**Figure 10:**
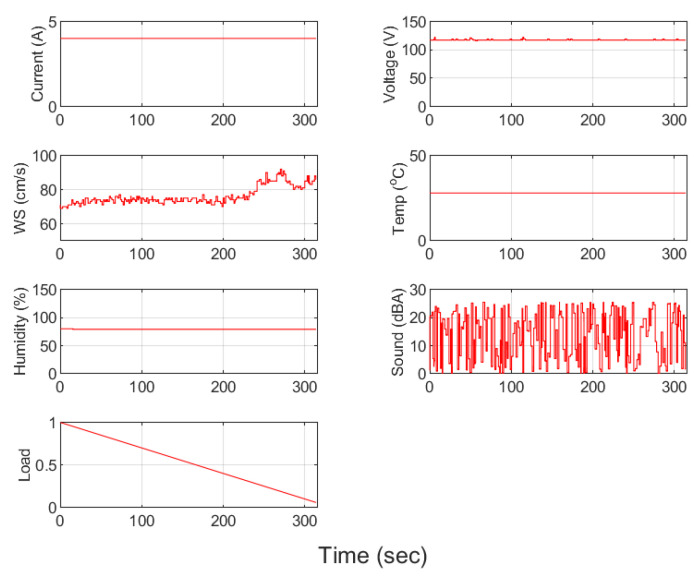
Samples of sensing data.

To verify the feasibility of the developed SMB, an air filtering platform is adopted for the predictive maintenance. As shown in Figure 9, the sensors of current, voltage, wind speed, temperature, humidity, and sound are considered. The capability of dust filtering is considered as the metric to identify the usability of the system. In the correlation analysis, as shown in Table 1, the load reflects the accumulation degree of the filter. The samples of sensing data through the smart machine box are shown in Figure 10, where the load represents the degree of accumulated dust. The accumulated dust becomes greater as the value of the load decreases. As more dust accumulates, it becomes more difficult for air to pass through the filter. Naturally, the wind speed (WS) will be affected, where the wind speed is the measurement from an anemometer. Through the correlation analysis, there exists the strongest relationship between the load and wind speed. 

**Figure 11:**
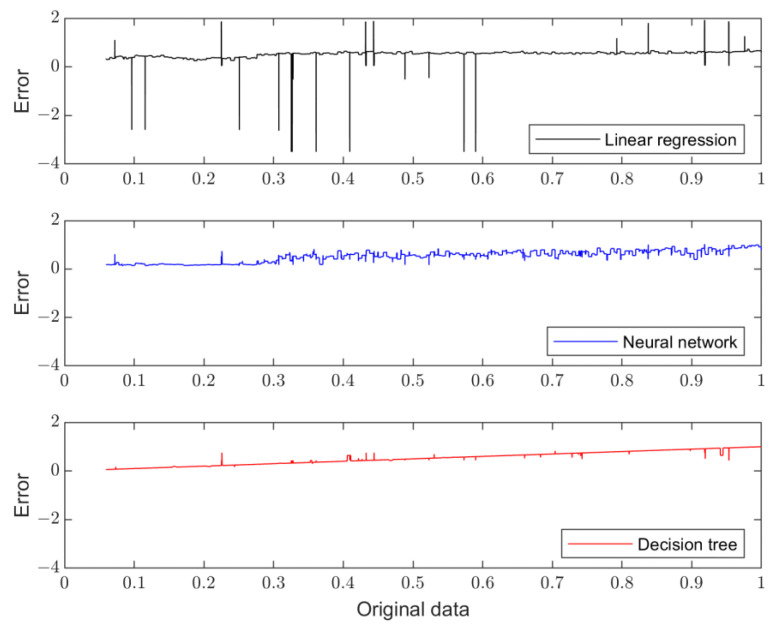
Model validations with different learning methods.

In the process of modelling and validation, three learning methods, linear regression, a neural network, and a decision tree, are considered. Linear regression is a supervised learning method, suitable for a regression analysis for prediction and forecasting. A multi-layer neural network (MLNN) is often used for supervised learning tasks, such as classification and regression. The main difference between the linear regression and neural network is that an MLNN is a more flexible model that can handle non-linear relationships, while linear regression is a simpler model that assumes a linear relationship between the variables. On the other hand, decision trees perform well on high-dimensional data sets. Decision trees can capture non-linear relationships between variables, which makes them suitable for a wide range of problem types, including classification and regression tasks. The difference between the real data and the model outputs of different learning methods is shown in Figure 11. The decision tree can provide the least validation errors among different modeling methods, shown in Table 2.

**Figure 12:**
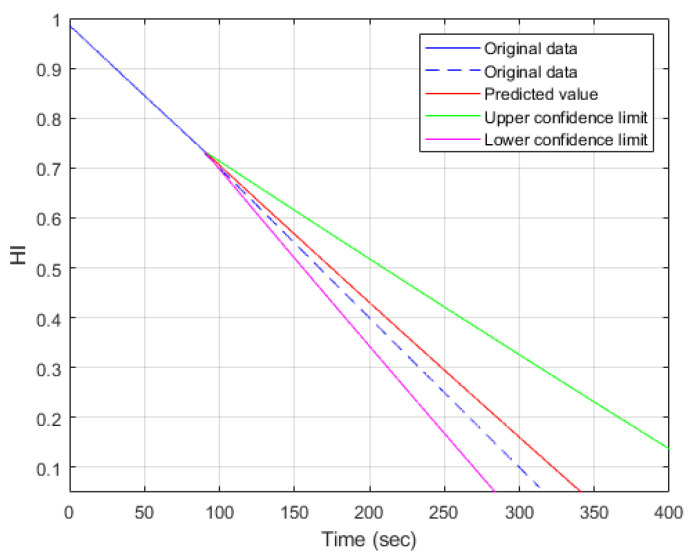
RUL prediction using decision tree with less history data (N = 4500).

**Figure 13:**
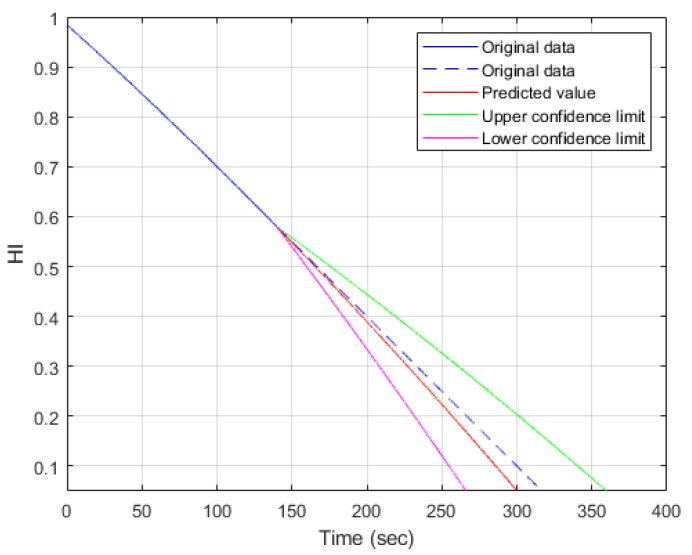
RUL prediction using decision tree with more history data (N = 7000).

The trained decision tree model is applied for the prediction of remaining useful life. In the analysis results, shown as Figure 12 and Figure 13, the blue line is the history data used for prediction. From the starting point of prediction, the dashed blue line is the real data, and the red line is the prediction result. In addition, the green lines indicate the upper and lower limits of the 95% confidence interval. The difference between the prediction result and the corresponding real data can be viewed as an index to illustrate the reasonability of RUL prediction. A confidence interval is a way of quantifying the uncertainty that tells you how confident you can be about the prediction accuracy. In the predictive maintenances, two cases are considered, in which one has less history data and one has more history data. The data number of one complete recording cycle is about 15,600. In the following cases, the first 4500 and 7000 real data values are used for health status prediction, respectively. The cases with less history data are shown in Figure 12, and the case with more real data is shown in Figure 13. The span of the confidence of the interval in Figure 12 is much larger than the span of the confidence interval in Figure 13. It is reasonable that the prediction confidence is higher with more history data. The health indicator (HI) indicates the health status of the test system. The higher the value of HI, the better the health status. Let HIth be represented as the threshold of the health indicator. The health status becomes significantly worse if the health indicator is less than HIth. Without loss of generality, the threshold of HI is selected as 0.4. 

From Figure 12, the HI value at the end of the blue line is 0.73, and the corresponding time instance is 90 s. In addition, the time instances corresponding to the real data and predicted value are 200 and 209 s, respectively. In summary, the predicted remaining useful life is 119 s, and the RUL prediction error is 9 s. Similarly, in the 7000-data-value case, it starts to predict the health status at a time instance equal to 140 s. The predicted and real health statuses corresponding to 0.4 HI are 197 and 200 s, respectively. Thus, the RUI using prediction is 57 s, and the prediction error of RUL is 3 s. The error of predicted RUL with more history data is much less than that of less real data—3 s to 9 s. Moreover, from Table 3, the span of the confidence interval corresponding to 0.4 HI is much smaller—35 s to 75 s. Similarly, if the threshold of the health condition is 0.3, the corresponding analyses of RUL predictions are summarized in Table 4. As shown in Table 4, the prediction errors of RUL are 12 s and 5 s corresponding to less and more history data, respectively.

Time-based maintenance, often referred to as preventive maintenance (PM), is a strategy used in industries to ensure the continued reliability and performance of equipment. This approach involves conducting maintenance activities at predefined intervals or time intervals, regardless of the equipment’s current condition. To illustrate the maintenance effectiveness of PdM, two scheduled time intervals (Ts) are considered for the regular preventive maintenance, Ts = 30 s and 600 s, respectively, shown as Table 5. Based on the scheduled checkouts, the characters “P” and “F” represent for the system working normally and abnormally, respectively. In the case of HIth = 0.3 and Ts = 20 s, the system is working normally at the time instance 201 s. However, the system becomes abnormal before the next scheduled time of 240 s. Similarly, in the case of HIth = 0.4 and Ts = 60 s, the status is normal under the scheduled checkout at the time instance 210 s. However, the system becomes abnormal before the next checking at 240 s. In general, time-based maintenance is conducted according to a prespecified time schedule. However, the system failures cannot be identified in advance until the failures happen. Alternatively, predictive maintenance can predict the remaining useful life of automation machines based on the data analyses of sensing information. The whole process involves data preprocessing, a feature analysis, model training, and RUL prediction. If the collected sensing data are appropriate, the predicted model can be obtained from some learning algorithms. Considering the cost–effect regarding unexpected failures, predictive maintenance could be a better solution to maintain the operation effectiveness of factory automation.

## 4. Discussion

**Figure 14:**
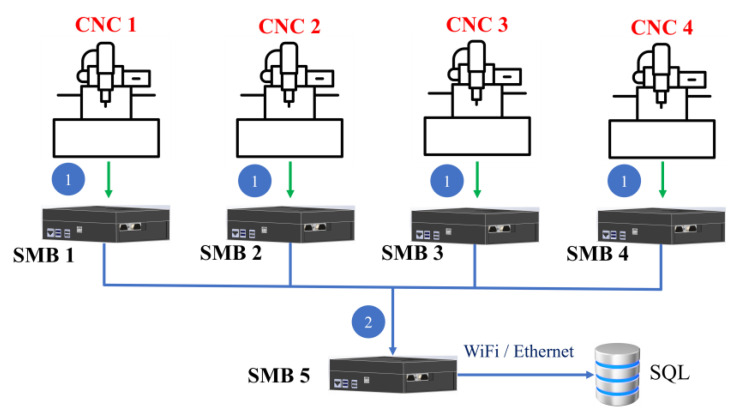
Automation machines operate in parallel (Blue 1 and 2 represent the first and second steps, respectively.).

**Figure 15:**
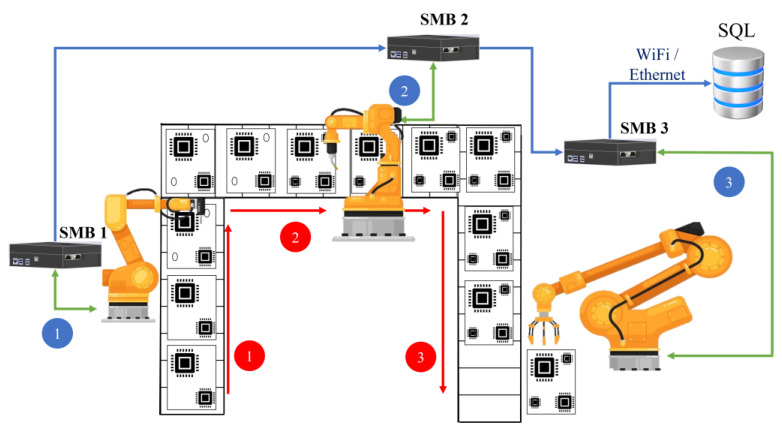
Automation machines operate in series (Blue 1, 2 and 3 represent the data collected in sequence. Red 1, 2 and 3 represent the working flow in sequence.).

A smart machine box was developed for gathering sensing information, whether from sensor modules or from automation machines. To achieve the goal of smart manufacturing, the collected data will be analyzed to improve the production performance. The computation core of the proposed smart machine box is built with Arduino and Raspberry Pi. Based on the ROS platform, the developed SMB has the capability of real-time monitoring. The built-in communication interfaces include DIO, AIO, UART, RS232, RS485, etc. To attain flexibility, the communication interfaces can be easily changed. For example, RS422 communication can be adjusted to RS485 with some wire connections. In another case, a signal transmitted through RS232 is ready to be sent, but the SMB has no extra RS232 interface available. To solve this problem, the user can choose the DIO instead, where the RS232 may be first transferred to UART, then the UART can transfer to DIO. In the possible applications of an automation factory, the operational environment will encounter more automation machines. Also, the designated demands would be various. For example, machines may run on an individually operated basis. To gather all machine statuses, smart machine boxes in the first layer can be deployed in parallel, shown as Figure 14 One more host machine box can be applied to collect all information to send to the database. In addition, automation machines may operate in series. It is commonly seen in a production line, where each machine performs its unique task, and the goal can be completed sequentially. Under this circumstance, the machine status can be transmitted in sequence, shown as Figure 15. The proposed smart machine box has the superiority in flexibility and scalability. The investigation on predictive maintenance explores the possible impact on smart manufacturing such that reliability of automation machines can be improved. 

## 5. Conclusions

In this study, a smart machine box is developed for the application on predictive maintenance. The proposed SMB integrates a single-board microcontroller Arduino and a single-board computer Raspberry Pi to gather sensing information to transmit to the remote database. The proposed smart machine box has the advantages in flexibility and scalability. Based on the ROS platform, the real-time monitoring and hot swapping can be achieved. An air filtering experiment platform is adopted for integrating tests. The collected raw data are sequentially executed in the stages of data preprocessing, a feature analysis, and prediction on remaining useful life. The analysis results illustrate the reasonability and feasibility of the testing scenarios. The possible expansion to a certain scale application is also addressed. In the future, we may consider deploying a tiny machine learning framework in the developed smart machine box. Then, the machine box could be promoted as an edge computing device. The contribution to the IoT era is optimistically expected.

## Figures and Tables

**Table 1:** Correlation analysis.

	Current	Voltage	WS	Temp	Humidity	Sound	Load
Current	1	0.141	0.154	0.199	0.055	0.011	0.009
Voltage	0.141	1	0.513	0.043	0.073	0.089	0.011
WS	0.154	0.513	1	−0.054	0.012	0.041	−0.436
Temp	0.199	0.043	−0.054	1	0.126	−0.094	0.040
Humidity	0.055	0.073	0.052	0.126	1	−0.019	−0.044
Sound	0.011	0.089	0.041	−0.094	−0.019	1	0.050
Load	0.009	0.011	−0.436	0.040	−0.044	−0.050	1

**Table 2:** Model validation comparisons of different methods.

	Linear Regression	Neural Network	Decision Tree
MAE	0.188	0.105	0.006
MSE	0.055	0.019	0.001
RMS	0.235	0.1137	0.031

**Table 3:** RUL predictions with different history data (HIth = 0.4).

	Start to Predict		HIth = 0.4			
N	HI	Time (s)	Predicted (s)	Real (s)	Lower (s)	Upper (s)
4500	0.73	90	209	200	182	257
7000	0.58	140	197	200	184	219

**Table 4:** RUL predictions with different history data (HIth = 0.3).

	Start to Predict		HIth = 0.3			
N	HI	Time (s)	Predicted (s)	Real (s)	Lower (s)	Upper (s)
4500	0.73	90	245	233	210	308
7000	0.58	140	228	233	208	261

**Table 5:** Health status checking with scheduled checkout.

Real Data		Scheduled Checkout			
		HIth = 0.3		HIth = 0.4	
Time (s)	HI	Ts = 30 s	Ts = 60 s	Ts = 30 s	Ts = 60 s
30	0.90	P		P	
60	0.82	P	P	P	P
90	0.73	P		P	
120	0.64	P	P	P	P
150	0.55	P		P	
180	0.46	P	P	P	P
210	0.37	P		F	
240	0.28	F	F		F

## Data Availability

Not applicable.
